# Sex Differences in Cancer Cachexia

**DOI:** 10.1007/s11914-020-00628-w

**Published:** 2020-10-12

**Authors:** Xiaoling Zhong, Teresa A. Zimmers

**Affiliations:** 1 Department of Surgery, Indiana University School of Medicine, Indianapolis, IN USA; 2 Indiana Center for Musculoskeletal Health, Indianapolis, IN USA; 3 Research Service, Richard L. Roudebush Veterans Administration Medical Center, Indianapolis, IN USA; 4 Department of Biochemistry and Molecular Biology, Indiana University School of Medicine, Indianapolis, IN USA; 5 IU Melvin and Bren Simon Comprehensive Cancer Center, Indianapolis, IN USA; 6 Department of Otolaryngology—Head & Neck Surgery, Indiana University School of Medicine, Indianapolis, IN USA; 7 Department of Anatomy, Cell Biology & Physiology, Indiana University School of Medicine, Indianapolis, IN USA

**Keywords:** Sex characteristics, Animals, Humans, Cachexia/etiology, Cachexia/pathology, Neoplasms/complications

## Abstract

**Purpose of Review:**

Cachexia, a feature of cancer and other chronic diseases, is marked by progressive weight loss and skeletal muscle wasting. This review aims to highlight the sex differences in manifestations of cancer cachexia in patients, rodent models, and our current understanding of the potential mechanisms accounting for these differences.

**Recent Findings:**

Male cancer patients generally have higher prevalence of cachexia, greater weight loss or muscle wasting, and worse outcomes compared with female cancer patients. Knowledge is increasing about sex differences in muscle fiber type and function, mitochondrial metabolism, global gene expression and signaling pathways, and regulatory mechanisms at the levels of sex chromosomes vs. sex hormones; however, it is largely undetermined how such sex differences directly affect the susceptibility to stressors leading to muscle wasting in cancer cachexia.

**Summary:**

Few studies have investigated basic mechanisms underlying sex differences in cancer cachexia. A better understanding of sex differences would improve cachexia treatment in both sexes.

## Introduction

Cachexia is a wasting syndrome with progressive weight loss and skeletal muscle wasting as its prominent phenotypic feature; other phenotypic features include fat mass reduction, chronic inflammation, anorexia, and fatigue. Many chronic illnesses, including cancer, chronic heart failure, chronic kidney disease, and chronic obstructive pulmonary disease [1–4], are associated with cachexia. Even some acute conditions such as sepsis and burn often end up with cachexia in the post acute phase [5–7]. Patients with cachexia suffer from impaired physical capacity, reduced emotional and social well-being, poor quality of life, and increased mortality [4, 8–10]. In the USA, the annual prevalence of cachexia in chronic illnesses is estimated to be over 160,000 hospital-admitted cases [11]; unfortunately, there are no approved, effective therapeutics to treat cachexia, despite many promising pre-clinical studies. Even more challenges exist for patients with cancer-associated cachexia given that chemotherapy per se can cause cachexia and that low skeletal muscle mass predisposes to higher chemotherapy toxicity and lower therapeutic response [12, 13].

While the impact of cachexia on cancer mortality has received increasing attention in recent years, sex differences in this syndrome are far less appreciated. Because men and women are biologically different, sex differences may manifest as different susceptibility to cachexia, different cachexia progression, or different response to therapeutic treatment. Understanding the mechanisms leading to such differences could permit tailored therapies as well as provide novel therapeutic insights. For example, any protective factors existing in one sex could potentially be used to lower the mortality in the opposite sex. Unfortunately, sex differences are sometimes noted but often not probed in clinical observational studies and are still not routinely evaluated in basic and translational research, with male animals predominantly used in experimental systems [14–16]. This review aims to highlight the sex differences in phenotypic manifestations recently documented in humans and mouse models of cancer cachexia and our current understanding of and speculation on the potential mechanisms accounting for the differences.

## Sex Difference in Cancer Cachexia Phenotypes

Because weight loss and muscle wasting are the prominent phenotypic feature in cancer cachexia, the consensus definition of clinical diagnosis of cachexia includes weight loss > 5% over the past 6 months, BMI < 20 kg/m^2^ and ongoing weight loss > 2%, or low skeletal muscle mass/sarcopenia defined by imaging criteria and ongoing weight loss > 2% [4, 17, 18]. Other phenotypes including muscle fiber size and type as well as muscle weakness have been considered in different studies, as described below.

Cachexia affects approximately 50% of all cancer patients [19–21]. However, cancer cachexia, like most research areas, has primarily been studied in males and relatively few publications are available on sex differences [22]. Baracos et al. evaluated body composition in non-small cell lung cancer by analyzing diagnostic computed tomography (CT) images of 441 patients (229 men and 212 women); they reported that a much higher proportion of men (61%) than women (31%) showed muscle depletion (sarcopenia) [23]. As well, in a study of 190 cancer patients, 88% with gastrointestinal malignancies, prominent sexual dimorphism was observed in *rectus abdominis* CT cross-sectional area, mean fiber cross-sectional area, and expression of genes associated with atrophy (FOXO1), muscle growth (AKT1, MSTN, etc.), apoptosis (CASP9), and inflammation (TNF and STAT3) [24]. Wallengren et al. obtained a similar result in a study of 471 cancer patients (259 men and 212 women) where the prevalence of muscle depletion in the last 2 years of life was higher in men than women (59% vs. 28%) [25]. In a study of a large cohort of hospitalized patients with various types of cancers (*n* = 597) or benign disease (*n* = 903), Norman et al. showed that reduction of grip strength in patients with severe weight loss was greater in men than women [26]. Stephens et al. studied 35 males and 19 females with gastrointestinal cancer (esophageal, gastric, pancreatic, bile duct, rectal), among which 15 males and 9 females were classified as cachectic based on weight loss ≥ 10%. They observed that male patients with cachexia had reductions in low limb muscle mass, strength, power, and muscle quality compared with controls, whereas only two of these measures including muscle strength and muscle quality were reduced in female patients with cachexia (defined here as greater than or equal to 10% body weight loss) [27]. Furthermore, they observed that decreased mechanical quality associates significantly with a decline in subjective quality of life measures in males, but not in females. A study of 84 patients with advanced cancer (48 men and 36 women) showed a strong association between muscle mass and cancer-related fatigue in males, but no similar relationship in females [28]. Among experimental studies, male mice lose a greater percentage of body weight than female mice in the *Apc*^Min/+^ colorectal cancer model of cachexia [29].

The impact of cancer cachexia on clinical outcomes has been evaluated in males versus females. For example, Burkart et al. [30] investigated a cohort of 109 patients with aggressive B cell lymphoma. They reported that both progression-free survival (PFS) and overall survival (OS) were decreased in males with sarcopenia compared with those without and that males with adipopenia had decreased OS compared with the non-adipopenic group. However, in females with sarcopenia, there was no difference in PFS and there was a trend for improved OS, and there was no difference in the PFS and OS in adipopenic versus non-adipopenic women.

It is well known that cancer is an inflammatory disease [31] and the severity of inflammatory diseases strongly correlates with the super-induction of proinflammatory cytokines. Indeed, cancer-mediated systemic inflammation is the driving force of muscle wasting in cancer cachexia [32, 33]. In the study conducted by Wallengren et al. as cited above, patients with increased C-reactive protein have less muscle mass and lose muscle mass at an accelerated pace during the disease trajectory [25]. It is well documented that males and females respond to inflammation differently [29, 34–36]. Unsurprisingly then, sex differences exist in cancer cachexia progression and inflammation [29, 37–39]. For example, high serum proinflammatory cytokines including interleukin-6 (IL-6) and IL-6 family cytokines IL-11, LIF, and Oncostatin M associate with cachexia in Colon-26 (C-26) tumor-bearing female mice and IL-6/STAT3 activation in skeletal muscle induce both acute phase protein synthesis and skeletal muscle wasting [40]. IL-6 receptor antibody treatment blocked cachexia progression through the suppression of muscle protein degradation in male *Apc*^Min/+^ mice [41]. However, this group showed that unlike the male, significantly higher plasma IL-6 levels in female *Apc*^Min/+^ mice electroporated with an IL-6 overexpression plasmid than with a control plasmid did not induce or accelerate cachexia progression [29]. In terms of the mechanisms accounting for the differential sex response to IL-6 treatment, the authors seemed to exclude the possibility that differential muscle gp130 and IL-6 receptor expression would be the cause. Instead, the downstream regulators such as STAT3 and SOCS3 that have the potential to alter muscle IL-6 signaling [39, 42] may be in a differential expression or activation state between males and females. Regardless, differential sex regulation of cancer cachexia progression might lead to differential response to the therapeutic treatment.

## Sex-Related Muscle Fiber Composition in Cancer Cachexia

In skeletal muscle, mice have four major fiber types based on speed of contraction, fatigue, and metabolic properties: type 1 (slow oxidative), type 2A (fast-twitch oxidative glycolytic), type 2X (intermediate between 2A and 2B), and type 2B (fast-twitch glycolytic); while humans have three types (slow type 1 and fast 2A and 2X) [43]. Slow fibers are specialized to support long-lasting contractile activity and fast fiber function in quick and powerful work. Fiber function reflects different muscle fibers’ metabolic state; type 2 fibers typically have lower oxidative capacity and rely on glycolysis and phosphocreatine to generate energy, leading to accumulation of fatigue-inducing hydrogen (H^+^) ions and inorganic phosphate (P_i_) during contraction [43–45]. Fiber type differences exist between males and females. For example, muscles in men generally have a greater abundance of type 2 fibers and are less resistant to fatigue than women [43, 44, 46–48].

While muscle fiber type is sexually dimorphic under physiological conditions, it is still largely undetermined whether such differences directly affect the susceptibility to stressors such as cytokine stimulation in cancer cachexia. However, different fiber types indeed display different susceptibility to cancer cachexia. For example, type 2 fibers were more prone to cancer-induced muscle loss than type 1 fibers in male mice with Colon-26 tumors [49], but in another study using female mice bearing C-26 tumor, type 1 fibers were more sensitive to cancer-induced wasting and this slow type 1 fiber atrophy was accompanied by an increase in fast type 2B [50]. Because these two studies using the same C-26 model gave rise to opposite results regarding which type of fibers, type 1 or type 2, was more sensitive to cancer-induced muscle loss but they did have used opposite sex host mice, an open question is whether this atrophic fiber type selectivity actually reflects sex effect. Interestingly, no selective fiber atrophy has been observed in a study of human patients with upper gastrointestinal or pancreatic cancer (30 men and 11 women) [51]. In a recent study [52] linking the RNA-binding protein, HuR, to fiber type specification demonstrates that while type 1 fiber-rich soleus muscle of male wild-type mice bearing the Lewis lung carcinoma was significantly wasted, muscle in the muscle-specific HuR knockout mice was preserved. The mechanism underlying the protective effect is through enrichment of type 1 fibers by HuR collaborating with the mRNA decay factor KSRP to destabilize PGC-1a mRNA. Regardless of lack of clarity on sexually dimorphic fiber types in cachexia, the study establishes that fiber type-specific factors may be targeted to overcome cancer-associated muscle wasting. Thus, it is necessary to understand the molecular mechanisms responsible for fiber type specification and their differential responses to atrophic inducers as well as the sex differences in these aspects.

## Sex Differences in Muscle Mitochondrial Metabolism

Mitochondrial function is one of the greatest contributors to whole body energy expenditure, and it is central to the metabolic homeostasis of skeletal muscle. Mitochondria are known as the powerhouse of the cell, oxidizing nutrients to generate high-energy ATP molecules that can be utilized by the cell to sustain energy-demanding processes including macromolecule synthesis, muscle contraction, active ion transport, and thermogenesis. Beyond the bioenergetic function, mitochondria have been increasingly recognized to have other important roles including generation of precursors for biosynthesizing macromolecules and serving as signaling organelles [53–57]. Through these important roles, mitochondria participate in maintaining the cell’s homeostasis and coordinating cellular adaptation to stressors such as nutrient deprivation, oxidative stress, and endoplasmic reticulum stress. As such, any factors including genetic and environmental ones, such as mitochondrial gene mutations or systemic inflammation, that disrupt mitochondrial functions would result in or worsen diseases and pathologies including cancer cachexia [57–61].

Mitochondrial alterations including disruption of mitochondrial morphology, dysfunctional autophagy, and increased apoptosis have been reviewed in cancer-induced muscle wasting [59, 60, 62–64]. The consequences of mitochondrial dysfunction include not only reduced ATP production but also increased reactive oxygen species (ROS) levels, leading to oxidative stress and damage to cellular proteins, lipids, and DNA. Of note, oxidative stress is induced by the loss of normal redox equilibrium, and thus, a decrease in antioxidative species can also cause oxidative stress [64–68]. Oxidative stress is thought as an atrophic mechanism that can modulate other mechanisms. For example, the ubiquitin proteasome system (UPS), calpains, or autophagy-lysosomal system are upregulated by ROS, while the anabolic pathway is inhibited by ROS [64, 68–71]. Brown et al. [72] examined mitochondrial degeneration during the progression of cancer cachexia in two male mouse models, the Lewis lung carcinoma implantation model and the genetic *Apc*^*Min/+*^ colorectal cancer model. This study demonstrated that functional mitochondrial degeneration is an early event prior to muscle wasting in the development of cancer cachexia in both models. This important observation provides a rational for early intervention of cancer cachexia. Although it is established that mitochondria are functionally altered in cancer cachexia and contribute to the development of cachexia, sex differences in mitochondrial dysfunction in cancer cachexia are less addressed. However, sexual dimorphism of mitochondria in cardiac and skeletal muscles has been reviewed [73]. Cardinale et al. [74] reported significant novel sex differences in skeletal muscle mitochondria showing that women have higher intrinsic mitochondrial respiration and higher mitochondrial oxygen affinity than men with similar mass-specific mitochondrial respiratory capacity, suggesting that women possess superior mitochondrial quality relative to men. Another study [75] demonstrated that men and women have similar maximal respiration rates but different substrate sensitivity with women having lower mitochondrial ADP sensitivity and greater sensitivity to malonyl-CoA-mediated respiratory inhibition. Together these baseline sex differences provide the foundation for studying the role of mitochondrial bioenergetics within the context of metabolic perturbations and diseases. How much of these differences correlate with the differences in muscle fiber types between women and men, as discussed in the previous section, and whether these sex differences contribute to different susceptibility to cancer cachexia remain to be investigated.

## Sex Differences in Muscle Global Gene Expression and Cancer Cachexia

A central element in the study of molecular mechanisms underlying normal or disease conditions is to characterize the complete set of transcripts or proteins encoded by the genome of an organism, termed global transcriptome and proteome respectively. Of note, doing both omics may provide additional insights because oftentimes the accordance between an mRNA and the cognate protein is low. Skeletal muscle is the largest tissue in the human body with very active metabolism and as such is among the greatest contributors to whole body energy expenditure. To provide a reference for investigation of diseases, Lindholm et al. used deep RNA sequencing to investigate the global baseline transcriptome of 48 skeletal muscle biopsies from 18 resting humans (9 females and 9 males) with 6 of 18 contributing 2 biopsies from each leg and the remaining 12 contributing 1 from each leg [76]. One finding was the profound transcriptomic difference between men and women with > 3000 differentially expressed genes and > 5000 isoforms. Interestingly, they found a difference in oxidative metabolism-related pathways—mitochondrial function-related genes were enriched in females, while protein catabolism-related genes were enriched in males. In addition, consistent with the connection of oxidative type 1 fibers with a higher capillary density, several endothelial markers were enriched in females. Moreover, not only does sex dimorphism exist in the human muscle transcriptome but the difference also exists in the epigenome of human muscle and muscle-derived myoblasts and myotubes [77]. Whether such differences exist in the muscle in response to cancer in conditions of cancer cachexia are yet unknown.

The above discussed sex differences in global transcriptome and epigenome of skeletal muscle may be responsible for the differences in physiological characteristics such as muscle oxidative vs. glycolytic fibers, fatigue-resistant vs. fatigue sensitive fibers, or different mitochondrial quality, but future work is needed to determine how much they contribute to cancer cachexia susceptibility. In rodent models, it has been repeatedly reported that cachectic vs. non-cachectic tumor-bearing mice have distinct global gene expression profiles in skeletal muscle with dysregulation of critical pathways including inflammation, protein ubiquitination, and mitochondrial dysfunction [40, 78]. These studies were performed in either males or females; whether such pathways are different between the sexes in mice with cancer cachexia is also thus far undescribed.

## Sex Hormonal vs. Sex Chromosomal Role and Cancer Cachexia

Sex differences can be fundamentally attributed to hormonal and chromosomal effects. The former effect has been rather thoroughly investigated, although not explicitly in cancer cachexia. The estrogens, also referred to as female sex hormones, through the estrogen receptor-mediated signaling have been shown to exert protective effects on skeletal muscle mitochondrial biogenesis [73], muscle mass [79], regeneration [80], and satellite cell growth [81]. In addition, they can reduce age-related increases in pro-inflammatory cytokines [82] that otherwise may cause muscle loss through increasing muscle protein degradation [83]. However, there are limited data available on the chromosomal effect. To clarify whether there is any aspect of sex difference that can be attributed to chromosomal rather than hormonal differences, Penaloza et al. [84] cultivated cells, taken from male and female whole mouse embryos at ED10.5 when the gonadal development has not initiated, kidney at ED17.5 after the first embryonic assertion of sexual hormones, and kidney at postnatal PN17 (puberty). The sex of the ED10.5 embryos, which are anatomically undifferentiated, was determined by PCR amplification of sex chromosome-linked genes in the tail tissues. After treatment with stressors, the cells responded to stressor-induced cell death in a sex- and developmental stage-modulated manner. Female cells are significantly more sensitive than male cells, and ED10.5 embryonic cells show the largest sexual difference. The sex difference in ED10.5 embryo cells clearly indicates that chromosomal differences can by themselves generate differences in cell behavior. Whether sex chromosomes specify dimorphic response in cancer cachexia is unknown, however.

Because differentially expressed miRNAs have been associated with metabolic alterations, inflammatory responses, and cancer-induced muscle wasting in humans and rodent models and the molecular targets for these miRNAs are involved in the regulation of catabolism, acute phase response, and muscle degenerative or regenerative capacity [85–89], we wondered whether sex-related factors such as sex chromosomes-linked genes would be the source of differences in the roles of altered miRNAs expression in cancer cachexia. However, no publication has addressed this in cancer cachexia. To begin addressing the possibility, we investigated the validated cachexia-associated miRNAs reported in the literature [86, 88] to see whether any of the miRNAs are located on X chromosome by aligning them with the miRNA genes listed in the miRBase [90] (mirbase.org). The alignment indeed identified several on X chromosome, including mir-106a, mir-221, mir-223, mir-362, mir-384, mir-424, mir-450a, and mir-450b, which are shown in Table 1 along with their target genes. Many of the targets have been well studied for their roles in cancer-induced muscle wasting, such as Stat3 and Fbox32. This trial investigation is encouraging; with more studies revealing alterations in miRNA expression and function in cancer cachexia, it is possible to identify more X-linked miRNAs. We hypothesize that the X-linked miRNAs contribute to the sex differences in cancer cachexia given the strong post-transcriptional regulatory power of miRNAs able to target 30–50% of all protein-coding genes. Interestingly, according to miRBase, there are high number of miRNA genes located on chromosome X, 118 at present in humans in comparison with 1917 in total on all chromosomes and 92 in mice in comparison with 1234 in total; in contrast, there are only 4 on human chromosome Y and none on mouse chromosome Y. One potential mechanism for the dysregulation of X-linked miRNAs could be through escaping inactivation. Approximately 15–30% of human X chromosome-linked genes escape inactivation (XCI), a mechanism that silences a randomly chosen X chromosome in females to ensure X-linked gene dosage compensation between females (XX) and males (XY). Because some X-linked miRNAs are intronic in the protein-coding genes [91] and some of the host protein-coding genes have been shown to escape X chromosome inactivation, certain miRNAs are likely subject to escaping XCI [91–94], leading to imbalanced or enhanced expression between sexes and to a sex-specific response. Of course, this novel mechanism we propose requires validation in the future by more studies profiling the expression of miRNAs in both males and females with cancer cachexia and analyzing the results by sex. As such, we hope to see our proposal would stimulate studies on the contribution of altered sex chromosome-linked genes to the sex differences in cancer cachexia.

**Table 1:** X chromosome-linked genes coding for cancer cachexia-associated miRNAs

miRNA gene ID	Target genes	Reference
mir-106a	Stat3, Mef2c	86
mir-221	Bnip3	86
mir-223	Stat3, Mef2c	86
mir-362	Nr3c1, Comp, Pck1	86
mir-384	Fbxo32	86
mir-424	FASN, SMAD3, VEGFA, SPI1, HIF1A, SMAD7, MAP2K1, FGFR1, CCND3, CCND1, SMURF1	88
mir-450a	STAT1	88
mir-450b	BID	88

## Conclusions

**Fig. 1:**
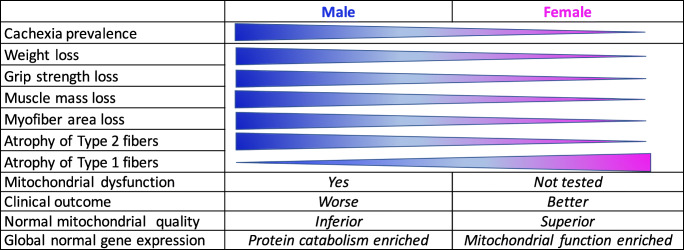
Summary of sex difference in cancer cachexia phenotypes. Shown are the observations with clearer sex differences. Italics, the difference is not conclusive or not documented in cancer cachexia

In this review, we highlighted the sex differences in normal skeletal muscle and cancer cachexia revealed by studies of humans and rodent models. Males and females display physiological differences in many aspects including muscle fiber types, muscle mitochondrial composition, and function, as well as muscle global gene expression patterns. Female muscles are more fatigue-resistant and have a superior mitochondrial quality than male muscles; mitochondrial function-related genes are enriched in females, while protein catabolism-related genes are enriched in males. In addition, there are potential sex differences in X-linked miRNAs escaping from inactivation. However, sex differences in cancer cachexia are largely limited to observations of clinical phenotypes. Male cancer patients generally have higher prevalence of cachexia, greater weight loss or muscle wasting, and worse outcomes compared with female cancer patients. In mouse models, response to the pro-inflammatory cytokine IL-6 may be subject to sex regulation during cachexia progression. These limitations are because most of previous studies have predominantly used males, and in some studies including both sexes, the data are combined in doing analysis. Overall, very few studies in cancer cachexia have explicitly investigated basic mechanisms for the sexual dimorphism. Thus, questions remain on how much of the physiological sex differences contribute to the difference in susceptibility to diseases such as cancer cachexia. Determination of the underlying molecular mechanisms would allow identification of targets for the development of tailored therapeutics. A summary for the above reviewed observations is presented in Fig. 1.
